# Estimation of Dermatological Application of Creams with St. John’s Wort Oil Extracts

**DOI:** 10.3390/molecules17010275

**Published:** 2011-12-28

**Authors:** Ivana Arsić, Ana Žugić, Vanja Tadić, Marija Tasić-Kostov, Dušan Mišić, Marija Primorac, Dušanka Runjaić-Antić

**Affiliations:** 1 Department of Pharmaceutical Research and Development, Institute for Medicinal Plant Research "Dr Josif Pančić", Tadeuša Košćuška 1, 11 000 Belgrade, Serbia; Email: azugic@mocbilja.rs (A.Ž.); vtadic@mocbilja.rs (V.T.); drunjaic@mocbilja.rs (D.R.-A.); 2 Department of Pharmacy, Faculty of Medicine, University of Niš, Dr Zoran Đinđić Boulevard 81, Niš, Serbia; Email: marijatk@medfak.ni.ac.rs; 3 Department of Microbiology and Immunology, Faculty of Veterinary Medicine, University of Belgade, Bulevar oslobođenja 18, 11 000 Belgrade, Serbia; Email: dusan@vet.bg.ac.rs; 4 Department of Pharmaceutical Technology and Cosmetology, Faculty of Pharmacy, Vojvode Stepe 450, 11 000 Belgrade, Serbia; Email: primorac@pharmacy.bg.ac.rs

**Keywords:** St. John’s Wort, oil extracts, sunflower, olive, palm oil, dermatological application

## Abstract

*Oleum Hyperici*, the oil extract of St. John’s Wort (SJW), is one of the oldest folk remedies, traditionally used in the topical treatment of wounds, bruises, ulcers, cuts, burns, hemorrhoids and also as an antiseptic. Considering the advantageous characteristics of emulsion applications, in the present study we have formulated three O/W creams containing 15% (w/v) of SJW oil extract as an active ingredient. The aim was to estimate dermatological application of the prepared creams for the abovementioned indications. The extracts were prepared according to the prescriptions from traditional medicine, however with different vegetable oils used as an extractant, namely: Olive, palm and sunflower oil. The investigated O/W creams demonstrated significant antiinflammatory effects in an *in vivo* double-blind randomized study, using a sodium lauryl sulphate test. Both skin parameters assessed in the study (electrical capacitance and erythema index), were restored to the baseline value after a seven-day treatment with the tested creams. Almost all investigated SJW oil extracts and corresponding creams displayed the same antimicrobial activity against the most of the investigated microorganisms with obtained minimal inhibitory concentrations values of 1,280 µg/mL, 2,560 µg/mL or >2,560 µg/mL.

## 1. Introduction

The oil extract of St. John’s Wort (*Oleum Hyperici*) is one of the oldest and most frequently used remedies in the folk medicine. Its reputation as a healer of wounds, bruises and ulcers, alone or as a part of a topical formulation, has been fairly well documented in the literature regarding medicinal plants [1,2]. According to Serbian folk herbals, it has traditionally been used in the topical treatment of cuts, burns, hemorrhoids and also as an antiseptic. It is prepared by maceration of the fresh flowering tops of *Hypericum perforatum* L. Hypericaceae (St. John’s Wort) in olive, sunflower or wheat-germ oil exposed to the sunlight for 40 days [3,4]. Moreover, the mode of preparation of *Oleum Hyperici* has been described in the supplement of German Pharmacopoeia [DAB 6] [5], while the British Pharmacopoeia suggest its usage in wound healing and the treatment of cuts and other skin and mucous membranes injuries [6]. In addition, German Commission E approved the topical application of *Oleum Hyperici* for the treatment and post-therapy of acute and contused injuries [7].

The traditionally claimed wound healing effects of *Oleum Hyperici* itself or in combination with other plants’ oil extracts has been proven by several scientific studies [8,9]. Part of this effect has been related to antibacterial activity [10]. Scientific data regarding the antibacterial activity of St. John’s Wort extracts prepared with various extragenses support their traditional usage for the treatment of wounds, skin and infectious diseases [11,12,13,14,15,16,17]. Some of the extracts, including the ethanolic and methanolic one, have been reported to exhibit more pronounced antibacterial activity against G-positive (G+) than G-negative (G−) bacteria [18,19,20,21]. However, Merel and Karabay claimed the same activity of methanolic extract against G+ and G− bacteria at concentration of 1,000 µg/mL [22]. Furthermore, an impact of solvent(s) and/or mode of preparation of St. John’s Wort extracts on their antimicrobial activity has been stated [22,23,24,25,26]. When it comes to an oil extract of St. John’s Wort (*Oleum Hyperici)*, the number of studies in the literature dealing with its antibacterial activity is limited. However, there are reports of antibacterial activity of *Oleum Hyperici* being formulated into topical preparations. A German patent describes an ointment containing olive oil extract of St. John’s Wort shortening the healing time of burns as well as showing antiseptic activity [17]. Peeva-Naumovska *et al.* reported antibacterial effects of lipophilic ointments containing 30–50% of *Oleum Hyperici* against five out of six investigated bacterial strains: *Streptococcus pyogenes* (two strains), *Streptococcus viridans*, *Micrococcus luteus* ATCC9341 and *Moraxella catarrhalis* [17]. A novel study of Süntar *et al.* demonstrated wound healing and antimicrobial activity of a topical formulation containing olive oil extract of St. John’s Wort, as well as sage and oregano essential oils [27].

Data concerning the chemical composition of *Oleum Hyperici* are controversial. It has been reported to contain hyperforin and its analogues as well as flavonoids (quercetin and I3, II8-biapigenin), but the absence of the naphthodianthrones (hypericin) is noted [28,29], although Isacchi *et al.* claimed only the presence of phloroglucinol derivatives and I3, II8-biapigenin [30]. Zdunić *et al.* confirmed the presence of quercetin and I3, II8-biapigenin [31]. In our previous study, quercetin, as well as total hypericins content was determined in three *Oleum Hyperici* samples prepared with different oil extragenses (*i.e.*, olive, palm and sunflower oil). Palm oil extract showed the highest quercetin content (21.68 μg/mL) as opposed to olive oil extract (5.8 μg/mL) with the lowest amount of this flavonoid. In contrast, palm oil had proved to be the worst solvent regarding hypericins extraction (0.001%), unlike olive oil and sunflower oil with three- (0.003%) and four times (0.004%) higher total hypericins content, respectively [32].

On the basis of the above mentioned results, it is not surprising that there are a number of preparations encompassing *Oleum Hyperici* for cutaneous application. For almost 30 years, our Institute has had its own product as a trade mark indicated for wound and burn healing and other injuries of the skin and mucous membranes, as well as for the treatment of dermatosis, hemorrhoids and myalgia. This product is a sunflower oil extract of fresh St. John’s Wort flowering tops.

Considering the advantageous characteristics of emulsion application which is reflected primarily in enhanced bioavailability of the active principle and the consequent increase of its therapeutic properties and spreading ability, in the present study we have formulated three creams containing *Oleum Hyperici* prepared with three different oil extragenses (*i.e.*, olive, sunflower and palm oil), as an active ingredient. Bearing in mind that most skin disorders are associated with more or less violated epidermal barrier of the skin, which is related to irritant reactions reflected by decreased skin moisture and/or increased skin erythema, the aim of our study was to evaluate the utility of formulated creams for the traditionally stated indications. Since irritant contact dermatitis (ICD) caused by chemical irritants is a non-immunological, inflammatory reaction, an evaluation of anti-inflammatory effect of the formulated creams was assessed in an *in vivo* double-blind randomized study, using Sodium lauryl sulphate (SLS), as a reference substance for experimentally induced ICD. Skin parameters observed were *stratum corneum* (SC) hydration level and erythema index (EI). In addition, *in vitro* antimicrobial activity of the prepared oil extract of St. John’s Wort and the corresponding creams was assessed against G+ and G− bacteria and yeast strains of dermatological relevance.

## 2. Results and Discussion

### 2.1. Chemical Characterization of Plant Material and Prepared Oil Extracts

**Figure 1:**
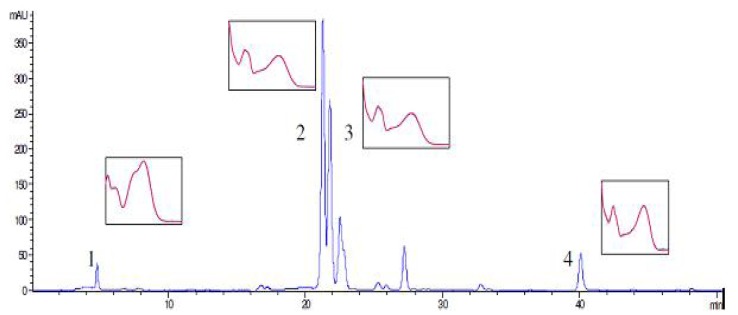
HPLC profile of SJW methanolic extract: (1) chlorogenic acid; (2) rutin; (3) hyproside; (4) quercetin.

HPLC analysis of methanolic extracts of SJW revealed the presence of phenolic compounds: rutin, hyperoside, quercetin, and chlorogenic acid (0.61; 0.78; 0.02 and 0.03%, respectively), as presented in Figure 1. As HPLC analysis of all investigated oil extracts confirmed only the presence of quercetin, we determined the quercetin content in *Oleum Hyperici* prepared according to the traditional medicine prescription with three different types of extragenses. However, its amount varied between samples. The lowest content of quercetin (5.8 μg/mL) was found in the sample prepared with olive oil (OO-E). In the sample SO-E, prepared with sunflower oil, amount of quercetin was 15.05 μg/mL. The sample prepared with palm oil (PO-E) showed the highest level of quercetin (21.68 μg/mL). 

Quantification of total hypericins in all investigated in SJW and SJW oil extracts (SO-E, OO-E and PO-E) was also carried out. The results are given in Table 1.

**Table 1:** Total hypericins content in SJW oil extracts.

*Sample*	*Hyp%*
SJW	0.47%
OO-E	0.003%
PO-E	0.001%
SO-E	0.004%

### 2.2. Stability Testing of Formulated Creams

*Centrifuge test*: No phase separation was observed either after 15- or 30-min centrifugation runs. 

*Appearance*: Samples were semisolid, white, shiny, leaving a homogenous and smooth smear on the glass plate. Upon the application on the skin, they formed a thin, non-stick, non-oily, non-occlusive film producing a pleasantly cooling feeling as a result of water phase evaporation. During 3 months of storage at different temperature (5 ± 2 °C, 22 ± 2 °C and 45 ± 2 °C) no phase separation was observed.

*pH values of creams*: There are insignificant changes in pH values of samples at all examined temperatures as a function of time. pH values of the examined samples were lower after preparation than those of the samples stored at 5 ± 2 °C, 22 ± 2 °C and 45 ± 2 °C. Insignificant changes of sample pH values as a function of time and storage temperatures indicated that the examined samples were stable. The data are summarized in Table 2. 

*Rheological characteristics*: Data are summarized in Table 3 and Table 4.

Remote changes in pH values and minimum and maximum viscosity, as a function of storage time and temperature, indicate that the creams prepared with 5% of emulsifiers and 15% of an oil extract remain stable during 90 days.

**Table 2:** pH values of the creamsstored at 5 ± 2 °C, 22 ± 2 °C and 45 ± 2 °C as a function of time.

Sample (storage T)	pH value		
	48 h after preparation	1 month after preparation	3 months after preparation
**C-OO-E (5** **±** **2** **°C)**	5.97	6.24	6.45
**C-OO-E (22** **±** **2** **°C)**	5.97	6.17	6.35
**C-OO-E (45** **±** **2** **°C)**	5.97	6.05	6.40
**C-PO-E (5** **±** **2** **°C)**	5.49	5.59	5.57
**C-PO-E (22** **±** **2** **°C)**	5.49	5.74	5.95
**C-PO-E (45** **±** **2** **°C)**	5.49	5.71	6.01
**C-SO-E(5** **±** **2** **°C)**	5.44	5.57	5.74
**C-SO-E (22** **±** **2** **°C)**	5.44	5.44	5.64
**C-SO-E (45** **±** **2** **°C)**	5.44	5.47	5.75

**Table 3:** Rheological characteristics/ samples stored at 45 ± 2 °C.

Sample	Maximum apparent viscosity η_max_ (Pas) (D = 10 s^−1^)		
	48 h after preparation	1 month after preparation	3 months after preparation
**C-OO-E**	3142	3454	3170
**C-PO-E**	3154	3178	3454
**C-SO-E**	3476	3544	3146

**Table 4:** Rheological characteristics/ samples stored at 5 ± 2 °C.

Sample	Minimum apparent viscosity η_max_ (Pas) (D = 10 s^−1^)		
	48 h after preparation	1 month after preparation	3 months after preparation
**C-OO-E**	2002	2116	2206
**C-PO-E**	2054	2304	2136
**C-SO-E**	1996	2067	2145

### 2.3. Antimicrobial Activity

Almost all investigated oil extracts and creams showed the same antimicrobial activity against most of the investigated microorganisms, with MIC values of 1,280 µg/mL, 2,560 µg/mL or >2,560 µg/mL. The strongest antibacterial activity of all oil extracts and creams (except cream C-SO-E, PO and SO) has been shown against *Bacillus anthracis*, with a MIC value of 320 µg/mL of SO-E, 640 µg/mL for OO-E and 1,280 µg/mL of all other oils, extracts and creams. C-PO-E demonstrated moderately strong antibacterial activity against *Streptococcus pyogenes*, *Corynebacterium pseudotuberculosis*, *Bacillus anthracis* and *Serratia rubidaea* strains, with a MIC value of 1,280 µg/mL. Oil extracts SO-E and OO-E, as well OO, also displayed moderately strong antibacterial activity against *Serratia rubidaea* strain with a MIC value of 1,280 µg/mL. Only C-PO-E inhibited growth of *Proteus mirabilis* at a concentration of 2,560 µg/mL, while other creams showed no activity against this bacterial species. Also, only C-PO-E inhibited growth of *Serratia rubidaea* at a concentration of 1,280 µg/mL. The investigated *E. coli* strains were not inhibited by the applied concentracions of oils, extracts and creams. All investigated oils, extracts and creams inhibited the growth of *Candida albicans* strains at a concentration at 2,560 µg/mL.

All investigated oils and extracts (except SO-E) showed activity with a MIC value of 2,560 µg/mL against investigated *Staphylococcus pseudintermedius* strain obtained from dog’s skin. For all other staphylococci included in the investigation, the tested oils, extracts and creams showed no antibacterial activity, with MIC values >2,560 µg/mL. Results obtained for the antimicrobial activity of SJW oil extracts in comparison to the corresponding vegetable oils were controversial. The addition of SJW to all the investigated vegetable oils led to lower MIC values only against * Bacillus anthracis*. Stronger antimicrobial activity of extracts compared to vegetable oils was observed for sunflower oil extracts against *Serratia rubidaea* and *Proteus mirabilis*, olive oil extract against *Streptococcus canis* and palm oil extract against *Klebsiella pneumoniae*. However, greater MIC values with the addition of SJW could be noted in the case of palm oil extract against *Streptococcus canis*, *Streptococcus pyogenes*, *Corynebacterium pseudotuberculosis* and *Serratia rubidae* andin the case of sunflower oil extract against *Staphylococcus pseudintermedius*, *Streptococcus pyogenes* and *Corynebacterium pseudo-tuberculosis.* The cases of stronger activity of creams compared to oils containing the same extract, *i.e.*, C-PO-E compared to PO-E(*S. pyogenes*, *C. pseudotuberculosis* and *S.rubidaea*) may be explained with greater sensitivity of mentioned strains to the emulsifiers used in the cream formulations.

The cases of stronger activity of oils with or without SJW compared to creams containing the same extract, *i.e.*, OO-E, OO and SO-E compared to C-OO-E and C-SO-E (*Bacillus anthracis*, *S.* *rubidaea*), might be explained by the greater sensitivity of the investigated strains to fatty acids from the oil.

Overall, the incoherent antimicrobial activity results of the plant extracts might be the consequence of inadequate contact of the extract with microorganism in liquid medium, which is frequently the case with oil extracts in water-based liquid media, no matter whether DMSO is used as a solvent.

The measured volumes of investigated oils, extracts and creams was very small and from the microbiological point of a view, MIC values of 1,280 µg/mL and 2,560 µg/mL might be interpreted as no or weak antimicrobial activity [17,33]. But, if considering the fact that SJW oil for external usage should be used directly, undiluted, the obtained MIC values of 1,280 µg/mL and 2,560 µg/mL in this case may be considered as strong antibacterial activity. 

Direct influence of undiluted oils, extracts and creams should be investigated on above mentioned bacterial strains and yeasts. The main problem is how to obtain the MIC value of oils, extracts and creams when all available methodology depends on water-based culture media, thus water-unsolved substances cannot be tested. Also, further investigations on the cytotoxic effects *in vitro* of oils, extracts and creams on cell lines in concentrations equivalent to the obtained MIC values, as well as clinical investigations are needed.

### 2.4. Anti-Inflammatory Effect on SLS-Irritated Human Skin

**Figure 2:**
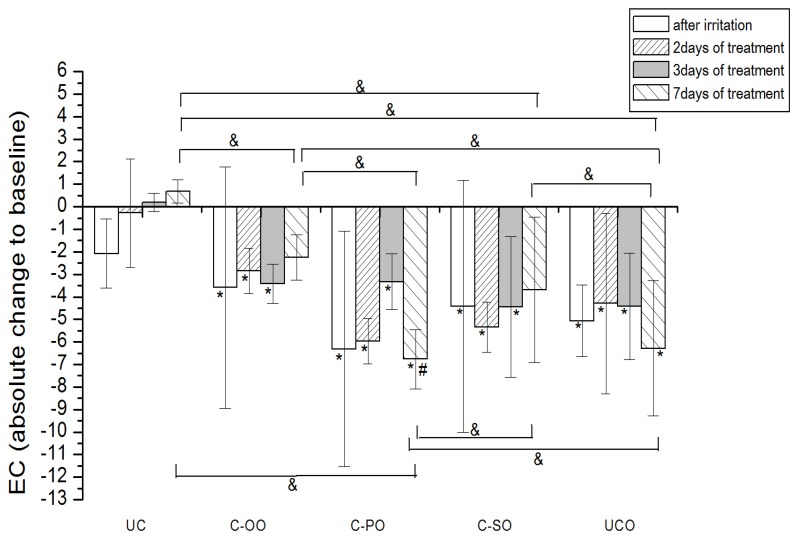
The influence of the irritation *per se* and samples C-OO, C-PO and C-SO after irritation on EC. Parameters were expressed as absolute changes to baseline at distinct time points. The effects of different formulations were compared mutually and to UC and UCO (significant differences being marked with &). Differences to corresponding sample with SJW (C-OO-E, C-PO-E and C-SO-E) were checked only on day 7, significant differences being marked with (#). (*) is for significant difference of EC at distinct time point related to baseline (means were compared).

**Figure 3:**
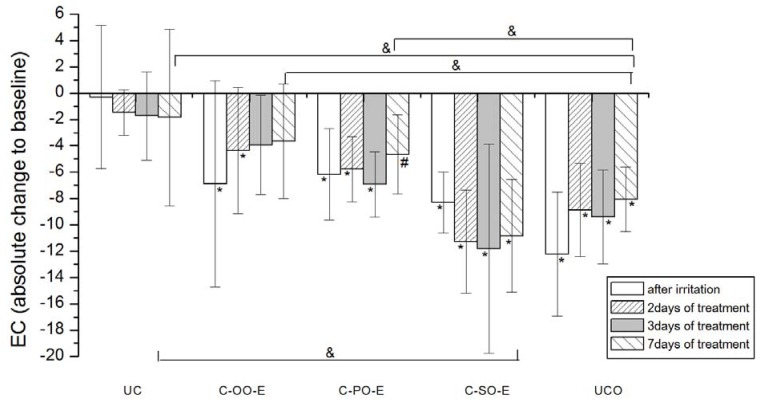
The influence of the irritation *per se* and samples C-OO-E, C-PO-E and C-SO-E after irritation on EC. Parameters were expressed as absolute changes to baseline at distinct time points. The effects of different formulations were compared mutually and to UC and UCO (significant differences being marked with &). Differences to corresponding sample without SJW (C-OO, C-PO and C-SO) were checked only on day 7, significant differences being marked with (#). (*) is for significant difference of EC at distinct time point related to corresponding baseline (means were compared).

**Figure 4:**
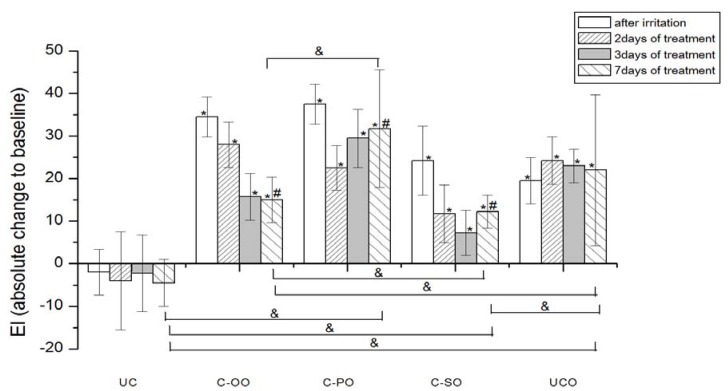
The influence of the irritation *per se* and samples C-OO, C-PO and C-SO after irritation on EI. Parameters were expressed as absolute changes to baseline at distinct time points. The effects of different formulations were compared mutually and to UC and UCO (significant differences being marked with &). Differences to corresponding sample without SJW (C-OO, C-PO and C-SO) were checked only on day 7, significant differences being marked with (#). (*) is for significant difference of EI at distinct time point related to corresponding baseline (means were compared).

**Figure 5:**
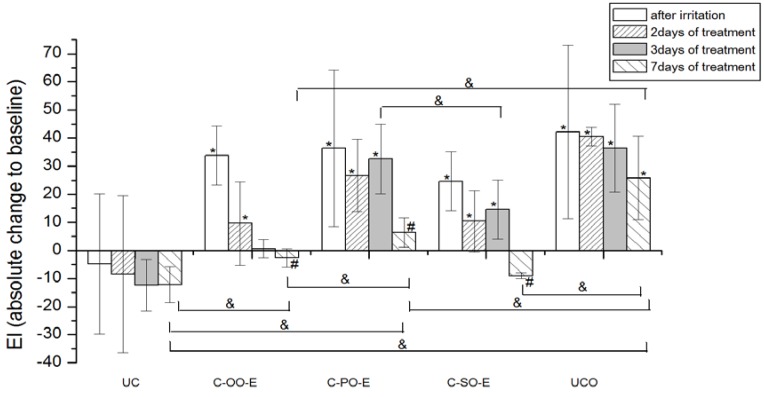
The influence of the irritation *per se* and samples C-OO-E, C-PO-E and C-SO-E after irritation on EI. Parameters were expressed as absolute changes to baseline at distinct time points. The effects of different formulations were compared mutually and to UC and UCO (significant differences being marked with &). Differences to corresponding sample without SJW (C-OO, C-PO and C-SO) were checked only on day 7, significant differences being marked with (#). (*) is for significant difference of EI at distinct time point related to corresponding baseline (means were compared).

Skin irritation is a complex biological event, commonly described by the clinical response (oedema, dryness, erythema) to chemical or physical stimuli that produces inflammation at the contact site after single or repeated exposures [34]. Exposure to surfactants often induces irritant contact dermatitis, which is a non-immunological local inflammatory reaction; sodium lauryl sulfate (SLS) is frequently used for that purpose [35]. Irritant contact dermatitis after the application of SLS is characterized by erythema, increased transepidermal water loss (TEWL), skin dryness and scaliness [36]. Although originally developed to estimate the susceptibility of substances to cause ICD, SLS test is often used to investigate barrier protective and anti-inflammatory features of topical preparations [37]. The results of the EC and EI measurements in SLS induced irritant contact dermatitis on human skin in a non-invasive *in vivo* model are presented in Figure 2 Figure 3 Figure 4 Figure 5.

#### 2.4.1. Electrical Capacitance

Occlusive application of 10% SLS resulted in a significant decrease in the skin hydration level compared to baseline values. (Figure 2 and Figure 3). The effects of the treatment using samples prepared with vegetable oils (placebo) on the irritated skin hydration level are given in Figure 2. Treatment with samples C-OO and C-SO led to a significant improvement of the skin hydration compared to baseline values after 7 days of application. On the other hand, application of sample C-PO did not show a significant difference related to corresponding baseline, even after 7 days of treatment. Nevertheless, level of hydration for the skin treated with this sample on day 7 (compared to the baseline value) was significantly lower than even in irritated untreated control (UCO). The effect of the oil type included in the cream formulation was clearly detectable, the sample C-OO showing the best moisturizing potential, yet with no significant difference compared to C-SO. As expected, in the comparison of the effect of the creams with vegetable oils at the different test sites, C-OO and C-SO showed significantly better hydration status of irritated skin compared to C-PO, as well as to the untreated control (UCO) after 7 days of the experiment.

As for the creams prepared with corresponding SJW oil extracts (active), significant improvement of irritated skin hydration level was noticeable after 3 days of treatment with C-OO-E and after 7 days of treatment with C-PO-E, while surprisingly there was no significant difference even after 7 days of treatment with C-SO-E related to corresponding baseline values. Again, sample prepared with olive oil (C-OO-E) showed the best moisturizing potential after 7 days of cream application, however with no significant difference in comparison to C-PO-E and C-SO-E.

Difference between the outcome of the treatment with the samples prepared with SJW oil extracts (active) *versus* the ones with corresponding vegetable oils was significant only in the case of palm oil (C-PO *versus* C-PO-E).

It is accepted that chemical irritants may cause skin irritation via two distinct pathways *i.e.*, via impairment of the barrier function of the SC and/or by direct effects on the cells of the keratinocytes of all epidermal layers [38]. The mechanism behind disruption of SC barrier by surfactants, especially SLS, include lipid bilayer disorganization, protein denaturation, increased water uptake by SC and swelling of the corneocytes [35]. Increase in the SC hydration has been reported after short-time exposure (e.g., 5 min) to SLS under occlusion, and has been related to structure disorganization leading to over exposed water-binding sites facilitating water fixation. However, this hyper-hydration is followed by sub-basal values of hydration, which is explained by incomplete repairment in SC lipid and protein structures and subsequent reduction in water binding capacity [39].

Our study revealed that seven-day treatment of SLS-irritated skin applying creams formulated with sunflower and olive oil led to the recovery of skin hydration level to the baseline values. However, the same treatment with cream containing palm oil as an oil phase did not demonstrate this effect. Although there was no statistically significant difference between the treatments with these two samples (*i.e.*, sunflower and olive oil) in the irritated skin hydration level, cream prepared with olive oil nevertheless displayed the best moisturizing potential. These results clearly indicate the impact of the vegetable oil used in the cream formulation. Differences between the oil type used and its influence on irritated skin hydration level could be explained by the differences in its chemical composition, *i.e.*, fatty acid composition.

Namely, in contrast to palm oil, sunflower and olive oil are replete with unsaturated fatty acids [40,41,42,43] and it is known that the degree of the fatty acid unsaturation is related to its fluidity [44]. Further, it has been shown that after application of emulsions on the skin, evaporation of the greatest percentage of water (evaporation phase) is followed by lipidization phase in which lipids from the emulsion penetrate into the epidermis and lead to increased skin hydration [45]. Therefore, it could be speculated that in the lipidization phase unsaturated fatty acids from the oil phase, owing to their good mobility, penetrate into the intercellular lipid lamellae much easier than saturated fatty acids [40,44,46] get incorporated in the SC and support or replace the endogenous fatty acids in the intercellular bilayers, thus repairing skin barrier and subsequently increasing the skin hydration [47]. Olive oil, as an oil phase of O/W emulsions, has been reported to have a positive influence on the increase of skin water content, however in a study investigating its effects on normal skin [48,49]. In addition, a direct role in barrier function of linoleic acid, a major sunflower oil component, has been proved [40,50].

The comparison in effects of the treatment with active *versus* placebo creams, revealed the significant difference only in the case of palm oil. The addition of SJW (C-PO-E) to placebo sample (C-PO) has had the clear impact on its ability to moisturize SLS-irritated SC, as seen in Figure 1 and Figure 2. These results indicate that chemical constituents of SJW extracted by palm oil play a role in the complex SC hydration recovery. However, further phytochemical investigations are necessary to provide more evidence in order to precisely determinate which constituent of SJW palm oil extract and in what manner influences this effect.

#### 2.4.2. Erythema Index

EI was significantly increased after SLS-induced irritation in both groups of participants at control sites (UCO) compared to the baseline values (Figure 4 and Figure 5).

None of the applied creams made with vegetable oils (*i.e.*, placebo) led to a significant EI decrease in comparison to corresponding baseline values in the seven-day treatment. However, when considering the EI transition in respect to baseline values, a significant difference after 7 days of the test has been noted between sample prepared with olive (C-OO) and both palm (C-PO) and sunflower oil (C-SO), as well as with untreated irritated control site (UCO).

On the other hand, significant EI recovery to the baseline values was observed after treatment with all the samples prepared with SJW oil extracts (*i.e.*, active samples), as follows: Sample prepared with olive oil SJW extract (C-OO-E) after 3 days and samples prepared with sunflower (C-SO-E) and palm oil SJW extract after 7 days of irritated skin treatment.

As expected, creams prepared with SJW oil extracts all pointed to significantly greater potential in decreasing EI of the irritated skin in comparison to corresponding creams prepared with vegetable oil (*i.e.*, C-OO-E *versus* C-OO, C-SO-E *versus* C-SO and C-PO-E *versus* C-PO) after 7 days of treatment.

When comparing effects of the creams prepared with SJW oil extracts at the different test sites, samples with olive (C-OO-E) and sunflower oil (C-SO-E) reduced EI of irritated skin significantly better compared to the sample with palm oil (C-PO-E), as well as to the untreated control (UCO) after 7 days of the experiment, however with no statistically significant difference between these two samples.

It is known that chemical irritants, in addition to disruption of SC barrier function, have a direct effect on skin cells. The later involves several mechanisms of inflammatory cascade initiation either via damaging keratinocytes membrane or by provoking reactive oxygen species (ROS) elevation to non-physiological level [38]. Erythema is considered to be the main feature of acute inflammatory reaction [51].

The results of our study demonstrated that all three SJW-containing creams offered a significant decrease of erythema to baseline values after a seven-day treatment, however with significantly better activity of samples prepared with SJW olive and sunflower extracts in relation to the one prepared with palm oil. Although the difference in EI reduction potential between these two samples themselves was not significant on the 7th day of an experiment, it was observed that the treatment with SJW olive oil cream resulted in earlier EI recovery (after 3 days of application) compared to SJW sunflower oil cream (after 7 days of application). In this regard, it could be stated that the cream with SJW olive oil displayed the best potential in EI recovery of experimentally irritated skin.

Olive oil has been reported to possess anti-inflammatory and oxygen scavenging effects. In experiments which included different topical assays regarding virgin olive oil, anti-inflammatory effect has been demonstrated and attributed to both unsaponifible and polar compounds while free radical scavenging has been thought to be due to the presence of polyphenols [41,52,53]. Moreover, topical application of olive oil has been described in treatment of several skin inflammatory conditions, including contact dermatitis [41]. However, in our experiments, placebo sample with olive oil (C-OO), as a part of an oily phase of O/W creams, failed to decrease EI of artificially irritated skin to the baseline value, after a seven-day treatment. On the other hand, significant difference after application of active (C-OO-E) *versus* placebo sample (C-OO) on the 7th day of the study, clearly emphasizes the influence of SJW addition. Hence, the assumption that phytochemicals of the SJW extract might be held accountable for this effect seems reasonable. Further chemical investigations are however necessary to prove more evidence and deeper insight in the mechanisms included in EI recovery.

## 3. Experimental

### 3.1. Materials

Samples of *Hypericum perforatum* L. were collected on Rtanj mountain, Serbia, during the flowering period, at the end of June 2009. A voucher specimen, No. 214/09, has been deposited at the Institute for Medicinal Plant Research "Dr Josif Pančić". Palm oil was purchased from Olitalia (Forlì, Italy), olive oil from Jan Dekker (Wormerveer, The Netherlands) and sunflower oil from Sunce (Sombor, Serbia).

### 3.2. Preparation of the Oil Extracts

Three oil extracts (OO-E, PO-E, SO-E) were prepared according to the prescriptions from traditional medicine *i.e.*, by maceration of 100 g fresh flowering tops of St. John’s Wort (SJW) in three different oils, namely OO-E with olive oil (OO), PO-E with palm oil (PO) and SO-E with sunflower oil (SO) and exposed to the sunlight for 40 days. Five hundred g of each oil extract was obtained [drug:extract ratio (DER) 1:5].

### 3.3. Chemical Characterization of Plant Material and Prepared Oil Extracts

Chemical characterization of the plant material as well as prepared oil extracts was carried out by HPLC, for identification and quantification of flavonoids (rutin, hyperoside, quercetin) as well as chlorogenic acid. HPLC analysis of St. John Wort and prepared St. John’s Wort oil extracts was carried out using the same method with Hewlett Packard HPLC model 1090; DAD detector (HP 1040); column Lichrospher RP-18 (5 µm, 250 × 4 mm i.d.) (Merck). The mobile phase A consisted of 99% H_2_O and 1% H_3_PO_4_, while B was acetonitrile. Flow rate was 1 mL/min, and elution was a combination of gradient and isocratic modes: 25–35% B, 0–2 min; 35–60% B, 2–8 min; 60–100% B, 8–12 min; 100% B, 12–25 min.

In the case of plant material, 1% extractive solutions in methanol were used, obtained through the water boiling reflux method for 30 minutes, in flasks with reflux coolers. Prior to injection, the samples were filtered through a 0.45 µm PTFE filter. The absorption was measured at 360 nm. The injection volume was 20 μL. Identification was carried out thanks to retention time and spectra matching. Once spectra matching succeeded, results were confirmed by spiking with the respective standards to achieve a complete identification by means of the so-called peak purity test. Those peaks not fulfilling these requirements were not quantified. Quantification was performed by external calibration with standards.

Samples for the HPLC analysis of St. John’s Wort oil extracts (SO-E, OO-E and PO-E) were prepared by further reextraction with methanol: A volume of each oil extract (50 mL) was reextracted three times with a portion of methanol (50 mL). The methanol extracts were combined, evaporated under vacuum at a temperature below 50 °C and concentrated to a volume of 10 mL. The samples were filtered through a 0.45 μm PTFE filter prior to injection. The absorption was measured at 370 nm. The injection volume was 20 μL. Quercetin was identified by co-injection method, using a commercial standard and its amount in investigated extracts was calculated using calibration curves. Concentrations used for calibration were 0.03–0.5 mg/mL. The results are presented as μg/mL of the oil extract.

The content of total hypericins in the plant material and prepared oil extracts was determined by spectrophotometric method, from three different probe and expressed in hypericin (% g/g). The extinction of the solution is read at a 587 nm wavelength, in a 1 cm tub, compared to appropriate solvent as a blank. The dosage of hypericin was established with the aid of absorbance measured at 587 nm, the quantity being calculated based on the formula for specific absorbance:
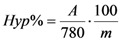

where A = the measured absorbance, m = grams of vegetable product in 100 mL of extract. 780 = specific absorbance of hypericine at 587 nm.

For the plant material sample preparation, 0.100 g of powdered St. John’s Wort was extracted for 30 min in boiling water to reflux with 100 mL of methanol in a flask with reflux coolers. After cooling, it is filtered and brought to 100 mL with methanol in a volumetric flask.

Samples for determination of total hypericins content in St. John’s Wort oil extracts (SO-E, OO-E and PO-E) were prepared by bringing 50 mL of the extract to 100 mL with chloroform in a volumetric flask. 

### 3.4. Formulation of O/W Creams

For obtaining the O/W emulsion-type cream, we used Emulgade^®^ SE emulsifiers [glyceryl stearate (and) Ceteareth-20 (and) cetearyl Alcohol (and) cetyl palmitate], cetearyl alcohol-Lanette^®^O, isopropyl myristate, caprilic/capric acid triglycerides (Myritol^®^318) all supplied by Cognis, Germany, Glycerol (Zorka, Serbia) and purified water according to Ph Eur 6.0. The concentration of SJW oil extracts and corresponding vegetable oils in all the creams was 15% (w/v). SJW oil extracts and corresponding vegetable oils used for the preparation of appropriate creams are given in Table 5. All the creams were preserved with potassium sorbate at a concentration of 0.15% (w/v). The samples were prepared using standard procedure for O/W emulsion-type cream.

**Table 5:** O/W creams prepared with different vegetable oils and corresponding SWJ oil extracts.

	Sample name	SJW oil extract	Vegetable oil
**Creams with vegetable oils (placebo samples)**	C-OO	-	OO
	C-PO	-	PO
	C-SO	-	SO
**Creams with SJW oils extracts (active samples)**	C-OO-E	OO-E	-
	C-PO-E	PO-E	-
	C-SO-E	SO-E	-

### 3.5. Stability Testing of Formulated Creams

*Centrifuge test*: The creams were centrifuged in two 15-min runs, each at 3,000 rpm (laboratory centrifuge: Tehtnica LC 320, Slovenia) 48 h after preparation. Phase separation was evaluated after 15 and 30 min.

*Accelerated stability testing*: The samples of creams were stored in thermostatic oven (Sutjeska, Yugoslavia) at 5 ± 2 °C and 45 ± 2 °C during three months after manufacturing.

*Long term stability testing*: The samples of creams were stored at 22 ± 2 °C during 3 months after manufacturing.

*Organoleptic properties*: Appearance, colour, odour and homogeneity of the samples were inspected both visually and by smear tests on glass slabs.

*pH values*: pH values of the creams were obtained by direct potentiometric method (pH-meter Hanna Instruments H8417, USA). Prior to the test, the instrument was calibrated by standard buffer solutions pH = 7.0 and pH = 4.0.

*Rheological characterisation*: Rotovisko RV 12 (Haake, Germany) with coaxial sensor cylinder systems SVst, Svdin, suggested in literature for semisolid systems examination, was used. All measurements were carried out at 20 ± 0.1 °C. The samples investigated were the ones stored at 5 ± 2 °C and 45 ± 2 °C.

### 3.6. In Vitro Antimicrobial Activity

The investigation of the antibacterial activity of three SJW oil extracts (OO-E, SO-E, PO-E) and the corresponding creams formulated (C-OO-E, C-SO-E and C-PO-E) was performed on Gram positive and Gram negative bacterial species–causative agents of skin infections in humans and animals. From the group of Gram positive microorganisms, *Staphylococcus aureus*, *Staphylococcus pseudintermedius*, *Streptococcus pyogenes*, *Streptococcus canis*, *Corynebacterium pseudotuberculosis*, *Bacillus anthracis* and *Listeria monocytogenes* strains were chosen*.* From the group of Gram-negatives, *Klebsiella pneumoniae*, *Pseudomonas aeruginosa*, *Escherichia coli*, *Serratia rubidaea* and *Proteus mirabilis* strains were selected*.* Pathogenic yeasts were also included in the investigation and two *Candida albicans* strains were chosen for that purpose. The investigated strains were isolated from skin swabs taken from the diseased persons and animals with skin infection symptoms, except two methicillin-resistant *Staphylococcus aureus* referential strains (MRSA ATCC 33591, MRSA ATCC 43300) and *Listeria monocytogenes* ATCC 19115 which were purchased from Becton Dickinson, USA. *Bacillus anthracis* was isolated from blood specimen taken from dead horse diagnosed with anthrax. The isolation was made from clinical material delivered to the Microbiology Department, Faculty of Veterinary Medicine, Belgrade University.

Conventional microbiological methods were applied for the purpose of isolation and identification and Columbia sheep blood agar (bioMerieux), MacConkey agar (bioMerieux), CNA agar with colistin and nalidixic acid (Becton Dickinson) and nutrient broth (BioLab) were used. For the isolation of *Candida albicans*, Sabouraud dextrose agar was used (BioLife). Identification of isolated strains was performed with BBL Crystal Gram-positive ID kit, BBL Crystal enteric/nonfermenter ID kit (Becton Dickinson), API 32 STAPH, API 20 NE and API 20 C AUX (bioMerieux).

For the investigation of antibacterial activity and the determination of MIC values of the prepared oil extracts and the corresponding creams, broth microdillution method was applied in accordance with the CLSI prescriptions for antimicrobial susceptibility testing [54,55,56]. The only modification of the method was the usage of oils instead of antibiotics. For that purpose, cation adjusted Mueller Hinton II broth was used (CAMHB, Becton Dickinson) with the addition of 1.6% bromocresol purple (Merck) in final concentration at 0.2 mL/200 mL for Gram-positives and 1% phenol red (Merck) at 1 mL/200 mL for Gram-negatives. Bromocresol purple and phenol red were added to obtain bacterial growth visibility. Sabouraud dextrose broth (BioLife) was used for yeasts with no indicators added. For streptococci, foetal bovine serum (Sigma) was added in CAMHB at final concentration at 5%. Dimethyl sulfoxide, (DMSO, Merck) was used as a solvent for both extracts and creams. For the complete dissolving of the creams, DMSO was heated in water bath on 60 °C until deposit of creams disappeared. Pure oils [*i.e.*, olive oil (OO), sunflower oil (SO) and palm oil (PO)] were used as controls. Investigated concentrations of oils, extracts and creams were 2560, 1280, 640, 320, 160, 80, 40, 20, 10, 5, 2.5 and 1.25 expressed in μg/mL. The oils were dissolved in DMSO at 25.600 μg/mL, then 1:10 dilution with CAMHB was made. Titration until desired concentrations was performed in microplate wells as previously described [54,55,56]. The final bacterial inoculum density of 5 × 10^5^ CFU/mL was achieved by adding 5 μL of 1–2 × 10^7^ CFU/mL suspension of investigated strain in microplate wells with 100 μL of previously added CAMHB. Microplates were incubated 18–24 h on 37 °C. For MIC values the broth with lowest oil concentration, with no visible bacterial growth, was used.

### 3.7. Anti-Inflammatory Effect on SLS-Irritated Human Skin

The present study was designed to evaluate and compare the effects of the creams containing different SJW oil extracts (C-OO-E, C-PO-E and C-SO-E) *vs.* creams containing corresponding vegetable oils (C-OO, C-PO and C-SO) against experimentally induced irritant contact dermatitis (ICD) on human skin. ICD, which can be evaluated by use of non-invasive biophysical measurements, was provoked using sodium lauryl sulfas (SLS) solution in a closed patch test [34,57]. Two parameters were measured: Electrical capacitance (EC) using the probe Corneometer® CM 825 to quantify the *stratum corneum* hydration (SCH) which can be affected by exposure to SLS; and erythema index (EI) using a Mexameter® MX 18 probe to quantify the erythema [36,57,58]. Both parameters were measured baseline, and after SLS induced irritation. Then, irritated skin sites were treated with specific test sample in order to study and compare the modification of this irritant reaction by different creams [57,58]. Both probes are the parts of the instrument Multi Probe Adapter MPA®9 by Courage & Khazaka Electronic GmbH, Germany.

The study was performed in a randomized, double-blind manner, in accordance with the Declaration of Helsinki and approved by the local ethics committee. A total of 20 female healthy volunteers (mean age 24 ± 3.2) was included after obtaining a written informed consent. Two places of the volar forearm of each arm were irritated as follows: 50 μL of 10% SLS water solution was pipetted on the piece of filter paper (3 cm × 3 cm) which was then attached to the skin, covered with Parafilm℘ and then with cotton adhesive tape. Baseline values were taken prior to the SLS application and the outcome 24 h after the occlusion, which lasted for 6 h, was removed. Then, the volunteers were divided in two groups; first group was instructed to apply samples C-OO, C-SO, C-PO and the second group applied the samples C-OO-E, C-SO-E, C-PO-E, in the morning and in the evening, each on one specific irritated skin site. Samples were marked with differently coloured labels. Two additional sites in both groups were left as a non-treated controls: irritated (UCO) or without irritation (UC). The measurements were also performed after 2, 3 and 7 days of the treatment. The effects of investigated samples were compared mutually, and related to both controls (UC and UCO).

All data were presented as mean ± standard error of the mean (SEM). *In vivo* measured parameters were expressed as absolute changes to baseline (Δ values); the values at distinct time points were compared to corresponding baseline values using paired sample t-test. The data from different sites (treated with different samples including both controls) at corresponding time points were compared using one-way ANOVA, followed by Tukey's t-test where appropriate. The values of measured parameters at distinct time points were compared to baseline values using paired sample t-test. Data obtained from skin sites treated with placebo *vs.* appropriate active cream (C-OO *vs.* C-OO-E, C-PO *vs.* C-PO-E and C-SO *vs.* C-SO-E) were compared using t-test for unpaired data after seven days of application. The differences were accepted as statistically significant at *p* < 0.05. Statistical analysis was performed with commercial statistical software package SPSS for Windows 17.0.

## 4. Conclusions

Significant anti-inflammatory effect of all the investigated O/W creams containing different SJW oil extracts was obtained in an *in vivo* double-blind randomized study, using SLS test. Both EC and EI, which served as readout parameters of SLS-induced skin irritation, were restored to the baseline value after a seven-day treatment with all the formulated creams. The exception was EC of the sample prepared with sunflower oil SJW extract. This minor discrepancy could be explained by the differences in individual skin physiological parameters and intrinsic complexity of hydration phenomenon itself, as previously described [59,60]. Almost all investigated SJW oil extracts and corresponding creams showed the same antimicrobial activity against the most of the investigated microorganisms with obtained MIC values of 1,280 µg/mL, 2,560 µg/mL or >2,560 µg/mL.
